# Early environmental enrichment and impoverishment differentially affect addiction-related behavioral traits, cocaine-taking, and dopamine D_2/3_ receptor signaling in a rat model of vulnerability to drug abuse

**DOI:** 10.1007/s00213-021-05971-z

**Published:** 2021-08-31

**Authors:** Lidia Bellés, Andrea Dimiziani, François R. Herrmann, Nathalie Ginovart

**Affiliations:** 1 Department of Psychiatry, Faculty of Medicine, University of Geneva, Geneva, Switzerland; 2 Department of Basic Neurosciences, Faculty of Medicine, University of Geneva, Geneva, Switzerland; 3 Division of Geriatrics, Department of Rehabilitation and Geriatrics, Geneva University Hospitals, Geneva, Switzerland

**Keywords:** Environmental enrichment, Environmental impoverishment, Dopamine release, D2/3 receptors, Impulsivity, Novelty preference, Cocaine

## Abstract

**Rationale:**

Risk factors for drug addiction include genetics, environment, and behavioral traits such as impulsivity and novelty preference (NP), which have been related to deficits in striatal dopamine (DA) D_2/3_-receptors (D_2/3_R) and heightened amphetamine (AMPH)-induced DA release. However, the influence of the early rearing environment on these behavioral and neurochemical variables is not clear.

**Objectives:**

We investigated the influence of early rearing environment on striatal D_2/3_R availabilities and AMPH-induced DA release in relation to impulsivity, NP, and propensity to drug self-administration (SA) in “addiction-prone” Roman high- (RHA) and “addiction-resistant” Roman low-avoidance (RLA) rats.

**Methods:**

Animals were reared post-weaning in either environmental enrichment (EE) or impoverishment (EI) and were assessed at adulthood for impulsivity, NP, and propensity to cocaine SA. EE and EI rats were also scanned using single-photon emission computed tomography to concurrently measure in vivo striatal D_2/3_R availability and AMPH-induced DA release.

**Results:**

EE vs. EI was associated with heightened impulsivity and a lack of NP in both rat lines. Higher dorsal striatal D_2/3_R densities were found in RHA EE and higher AMPH-induced DA release in RLA EE. Both impulsivity and NP were negatively correlated to dorsal striatal D_2/3_R availabilities and positively correlated with AMPH-induced DA release in EI but not in EE. EE vs. EI was related to a faster rate of cocaine intake and elevated active timeout responses in RHAs.

**Conclusion:**

Our results suggest non-monotonic, environment-dependent, relationships between impulsivity, NP, and D_2/3_R-mediated signaling, and suggest that EI vs. EE may decrease the reinforcing effects of psychostimulants in predisposed individuals.

**Supplementary Information:**

The online version contains supplementary material available at 10.1007/s00213-021-05971-z.

## Introduction

Drug addiction is a complex neuropsychiatric disorder characterized by high motivation for the drug, inability to refrain from drug-seeking, and compulsive drug-taking despite negative consequences (American Psychiatric Association 2013). Vulnerability to drug addiction results from the interaction of highly entangled factors, including genetics and environmental factors (Everitt and Robbins 2016; Kreek et al. 2005). A genetic predisposition to addiction is supported by a number of familial, adoption, and twin studies (review in Duaux et al. 2000). This genetic predisposition also manifests as certain personality traits such as impulsivity and sensation/novelty-seeking, which are both partly heritable (Sanchez-Roige et al. 2019; Wingo et al. 2016) and have both been identified as risk factors for addiction in rats and humans (Belin et al. 2016; Dalley et al. 2011; Ersche et al. 2010). More specifically, impulsivity and novelty preference in rodents have been associated with an increased susceptibility to develop compulsive drug self-administration (SA; Belin et al. 2011, 2008), while the former has also been related to the maintenance and escalation of drug SA (Anker et al. 2009; Dalley et al. 2007; Diergaarde et al. 2008) and increased propensity for relapse (Economidou et al. 2009). Moreover, studies in rodents support an association between impulsivity and novelty preference (Belles et al. 2020; Loos et al. 2009; Molander et al. 2011), suggesting that these two behavioral traits may be interrelated and could interact to increase individual susceptibility to develop addiction-related behaviors (Belin and Deroche-Gamonet 2012). The mechanistic link between impulsivity, novelty preference, and vulnerability to drug abuse may relate, at least in part, to abnormalities in striatal dopamine (DA) signaling such as a deficit in striatal D_2/3_ receptors (D_2/3_R; Dalley et al. 2007) and/or an exaggerated psychostimulant-induced DA release in striatum (review in Poulton and Hester 2020; Wingo et al. 2016). Besides genetic background, other factors, such as environmental factors, also play an important role in the etiology of substance use disorders. Consistent with this, exposure to positive environmental conditions during early life, such as good family and peer connectedness, higher socioeconomic status, and high level of education, may have protective effects and reduce the risk of addiction in humans (Kodjo and Klein 2002). In contrast, negative conditions such as a lack of social support during adolescence, parental neglect, or social exclusion can increase the risk of developing substance abuse later in life (De Bellis 2002). Environmental variables can also influence striatal DA function. For example, striatal D_2/3_R availability, which is inversely related to impulsivity (Belles et al. 2020; Dalley et al. 2007) and vulnerability to drug abuse (Dalley et al. 2007), can be altered by the social hierarchy in primates (Morgan et al. 2002) and is associated with social status and perceived social support in humans (Martinez et al. 2010). Available observations thus suggest that, in addition to genetic factors, potentially shared environmental variables may substantially influence drug abuse liability, impulsivity, and D_2/3_R-mediated DA signaling. The neurochemical mechanisms underlying the effects of environmental variables on vulnerability to drug abuse are however still unclear. Preclinical studies investigating the influence of the environment on the propensity to develop drug abuse frequently use the experimental paradigm of environmental enrichment (EE) in comparison with environmental impoverishment (EI). EE consists of exposing animals to physical, sensorial, and social stimulation, with physical exercise, social interaction, and exposure to complex and continuously changing stimuli greater than in EI, in which animals live alone without novel stimuli (Crofton et al. 2015). While EE has been shown to decrease the propensity to drug SA (Alvers et al. 2012; Bardo et al. 2001; Gipson et al. 2011), drug-seeking, and relapse (Chauvet et al. 2009; Thiel et al. 2009), it has also been shown to promote the development of compulsive drug-taking (Fouyssac et al. 2020). Evidence also indicates that EE affects impulsivity and novelty preference, although the direction of change is unclear, with studies showing that EE, relative to EI, can increase or decrease impulsivity (Dalley et al. 2002; Kirkpatrick et al. 2013; Perry et al. 2008; Zeeb et al. 2013) and novelty preference (Fernandez-Teruel et al. 2002; Rodriguez-Ortega et al. 2018; Vazquez-Sanroman et al. 2021; Zambrana et al. 2007). There are also studies suggesting that rearing conditions can affect D_2/3_R levels in the striatum, but the direction of changes, if any (Gill et al. 2013), is here again still unclear, with studies showing that socially enriched animals display either higher (Hall et al. 1998; Rilke et al. 1995) or lower (Djouma et al. 2006; King et al. 2009) levels of striatal D_2_R compared to socially isolated rats. It thus appears that environment variables can influence behavioral traits closely related to the propensity to drug abuse. More research is however needed to determine whether environment-related changes in striatal D_2/3_R availability and/or evoked DA release represent, at least in part, the molecular mechanism underlying an interaction between environment and predisposition to drug abuse.

The Roman high- (RHA) and low-avoidance (RLA) lines of rats represent a well-established model of vulnerability to drug abuse (review in Giorgi et al. 2019). When compared to RLAs, RHAs display higher levels of impulsivity and novelty-seeking (Belles et al. 2020; Moreno et al. 2010; Tournier et al. 2013) as well as a higher propensity to cocaine SA and to relapse following drug discontinuation (Dimiziani et al. 2019; Fattore et al. 2009). A growing body of evidence indicates that the Roman lines also display line-related differences in the functional properties of their DA neurotransmission system (review in Giorgi et al. 2019), such as differences in striatal D_2/3_R availabilities and in amphetamine- (AMPH-) induced DA release in striatum (Belles et al. 2020; Tournier et al. 2013). The goal of the present study was to investigate the influence of early rearing environment on impulsivity, novelty preference, and propensity to drug SA in the Roman rat lines. Moreover, as little is known on the effects of the rearing environment on indexes of striatal DA function, we also sought to yield a further understanding of the potential DA mechanisms underlying the effects of environmental variables on these addiction-related behavioral traits. To these goals, RHA and RLA rats were reared post-weaning in either EE conditions, with novelty, social interaction and exercise, or in EI conditions, where they lived alone without novel stimuli. Using a within-subject design, the effects of an early rearing environment were then assessed at adulthood on impulsivity, novelty preference, and propensity to cocaine SA. In an effort to further examine early rearing environment modulation of DA-dependent processes related to these traits, EE and EI rats were also assessed using single*-*photon emission computed tomography (SPECT) and the D_2/3_R radiotracer [^123^I]IBZM to concurrently measure in vivo striatal D_2/3_R availability and psychostimulant-induced DA release in relation to impulsivity and novelty preference.

## Material and methods

### Animals

A total of 56 male RHA and RLA rats from our permanent colony of outbred Roman rats at the University of Geneva were used. Rats were kept under a reversed 12-h light–dark cycle with lights off at 7:00 a.m., with controlled temperature (22 ± 2 °C) and humidity (50–70%). From the beginning and throughout the experiment, animals were food-restricted (85–90% of free-feeding weight), with water provided ad libitum. Experiments were performed in accordance with the Swiss Federal Law on animal care and were approved by the Animal Ethics Committee of the canton of Geneva.

### General procedure

Starting at weaning (postnatal day 21; PND21), rats were assigned to either environmental enriched (EE; *n* = 14 per line) or impoverished (EI; *n* = 14 per line) housing conditions where they remained for the entire experiment. Within 1 week following PND61, rats were tested for novelty preference using the novelty-induced place preference (NIPP) test. Subsequently, animals were trained in the five-choice serial time task (5-CSRTT) and, once the animals had acquired the task, they were tested for impulsivity. A subgroup of 40 rats (10 per group) was then scanned with SPECT and the D_2/3_R antagonist radiotracer [^123^I]IBZM to assesses their striatal D_2/3_R density and capacity to release DA in response to amphetamine (AMPH). Finally, the 56 animals were implanted with an intravenous catheter and exposed to cocaine SA for 14 days.

### Environmental housing conditions

The EE rats were housed in groups of 7 in large, stainless steel cages (122-cm width × 61-cm height × 46-cm depth), which were provided with climbing structures (ramps to second and third elevated levels), opportunities to shelter (houses, PVC tubes, cardboard tunnels), chewing materials (pieces of wood), a running wheel, and a set of 14 different non-chewable plastic toys and objects (balls, geometric figures, Legos). This set of toys and objects were rotated twice weekly, and all animals were handled daily. Rats in the EI condition were housed individually in standard type III cages (43 × 27 × 19 cm) with only one cardboard tunnel. EI rats were handled only briefly for scheduled bedding changes until the experimental protocol begins.

### Drugs

d-Amphetamine (Sigma-Aldrich, Switzerland) and cocaine (Pharmacy of the University Hospital of Geneva) were dissolved in saline. For perioperative care, amikacin (Bristol-Myers Squibb, Cham, Switzerland), cefazolin (Labatec Pharma, Meyrin, Switzerland), and buprenorphine (Reckitt Benckiser, Wallisellen, Switzerland) were diluted in saline.

### Novelty-induced place preference (NIPP)

Rats were tested for novelty preference in the NIPP test as previously described (Bardo et al. 1989). The apparatus consisted of four identical Plexiglas place-conditioning boxes (ActiMot, TSE Systems, Germany). Each box was comprised of two compartments (one designated familiar and the other novel), equal in size (48 × 48 × 40 cm), which differed in color, pattern walls, and floor texture. Both compartments were separated by a removable door. Rats were placed in the familiar compartment for 15 min once a day for 4 days. On the fifth day, the door between the familiar and novel compartments was open, and the animals were allowed to explore freely the two compartments for 15 min. The percentage of total time spent in the novel vs. familiar compartment was used as a measure of novelty preference.

### Five-choice serial reaction time task (5-CSRTT)

Methodological details on the 5-CSRTT are described in the Supplementary Information (SI). Each session began with the delivery of a reward pellet and the illumination of the house light and the food receptacle light. To initiate the trial, animals were required to nose poke into the food receptacle. After an inter-trial interval (ITI) of 2 s, a light stimulus located into one of the five nose-poke apertures was pseudorandomly illuminated for 30 s. A nose poke into the illuminated aperture was recorded as a correct response and rewarded with a pellet. A response into the wrong aperture (an incorrect response), a response during the ITI (a premature response), or no response within the 30-s limited hold (an omission) was punished with a 5-s timeout (TO) period during which all lights were turned off and no food was delivered. Throughout the training period, the level of difficulty progressively increased over 8 training phases, to reach the final goal parameters (1.5-s stimulus duration, 5-s limited hold, and 7-s ITI) and fulfill the criterion performance (> 80% accuracy and < 30% omissions) and stable baseline measures (< 10% variation in accuracy over 3 consecutive days). When animals achieved stable performance across 3 consecutive days (< 10% variation in accuracy), impulsivity was assessed by challenging the animals with a long ITI (9 s), a stimulus duration of 1.5 s, and a limited hold of 5 s. The session ended after either 100 trials or 60 min. Session length was increased to allow animals to complete all trials.

The performance measures analyzed were (Bari et al. 2008):

- Percentage of premature responses: [#premature responses/(#correct + #incorrect + #omission)] × 100, was used as a measure of impulsive action.
- The number of TO responses was a measure of compulsivity related to cognitive inflexibility.
- Percentage of accuracy: [#correct/(#correct + #incorrect)] × 100. Accuracy was taken as a measure of attention.
- Percentage of omissions: [#omissions/#trials] × 100. This was used as a measure of attentional function.

### *SPECT imaging and the radioligand [*^*123*^* I]IBZM*

Details on the radiotracer preparation, SPECT data acquisition, and analysis are provided in the SI. Under 2% isoflurane anesthesia, rats were injected i.v. with [^123^I]IBZM and scanned using the ultra-high-resolution multipinhole SPECT scanner (U-SPECT II, MiLabs, Utrecht, Netherlands) for a total duration of circa 134 min (67 frames of 2 min). At the beginning of the 36th frame (i.e., circa 72-min post-radioligand injection), rats were injected with AMPH (1.5 mg/kg: i.v.). The first (0 to 72 min) and second (73 to 134 min) portions of SPECT acquisition corresponded to the measure of [^123^I]IBZM kinetics at baseline and in response to AMPH, respectively.

Reconstructed SPECT images were processed using PMOD software v3.8 (PMOD Technologies Ltd., Zurich, Switzerland). SPECT images were coregistered to the MRI atlas of the rat brain (Schiffer et al. 2006). Transformation matrices were then applied to the SPECT dynamic images, mapping all rats into the same reference space. Regions of interest (ROIs) for the dorsolateral striatum (DST), the ventral striatum (VST), and the cerebellar cortex were defined on the MRI atlas, according to the rat brain atlas of Paxinos and Watson (1998). A ROI template was created that consisted of fixed-size circles (2-mm diameter) placed bilaterally on the DST and VST and a single ellipse on the cerebellar cortex. To minimize partial volume effects, ROIs were placed on the central planes in which the structures appeared. Time-activity curves (TACs) for the target-rich (DST and VST) and reference region (cerebellum) were extracted from dynamic images using a customized volume of interest template. TACs were analyzed using the linearized simplified reference region model (LSSRM; Alpert et al. 2003; Christian et al. 2006) to estimate the non-displaceable binding potential (BP_ND_) as an index of D_2/3_R availability and gamma as an index of AMPH-induced DA release in the striatum. Briefly, the LSSRM accounts for time-dependent changes in radiotracer binding (i.e., BP_ND_) induced by AMPH during the scan session. This is accomplished by introducing the time-dependent term gamma·h(*t*) in the model, where gamma represents the amplitude of radioligand displacement, and the function h(*t*) describes the timing of change following task onset. A positive gamma value is assumed to reflect the magnitude of [^123^I]IBZM binding reduction resulting from an AMPH-induced increase in DA release. The standard error of BP_ND_ and gamma as estimated by the nonlinear least-square fitting was expressed in percent of the parameter value (%SE) and used to assess the parameter identifiability (standard error, %SE; Carson 1986).

### Cocaine self-administration

Additional methodological details are provided in the SI. Rats were anesthetized with 2.0% isoflurane and implanted with an indwelling jugular catheter (Instech Laboratories, Plymouth Meeting, PA, USA) according to a procedure previously described (Dimiziani et al. 2019). Rats were then trained to self-administer cocaine (0.4 mg/kg/infusion) under a fixed ratio 1 (FR1) schedule during 2-h daily sessions for 14 consecutive days. At the beginning of the session, the house light was illuminated. Nose-poking of the rat into the active hole extinguished the house light and illuminate the stimulus light. Simultaneously, the infusion pump was activated and initiated an intravenous injection of cocaine at a rate of 0.1 ml in 4 s (0.06–0.1 ml, infused over 2.5–4 s, depending on the animal’s weight). The infusion was then followed by 20-s TO period during which both the house light and stimulus light were switched off. Further nose pokes into the active hole during TO had no consequence but were recorded. Inactive nose-poking was recorded but had no consequence. The position of the active and inactive holes was counterbalanced across rats. For ethical reasons, the total drug intake was limited to 60 infusions/session (24 mg/kg/session). Each session was terminated after 2 h or the delivery of 60 infusions. The criteria for acquisition of cocaine SA were (1) a mean of 35 cocaine infusions/session; (2) ≥ 70% of responses in the active hole; and (3) ≤ 15% variation in the number of infusions over two consecutive daily SA sessions. These criteria were adapted from previous studies (Carroll and Lac 1997; Rocha et al. 2005).

### Statistical analysis

Assumptions for normality of data distribution were verified using the Shapiro–Wilk test and non-normal data was log10-transformed. Data were subjected to two-, three-way, or mixed factorial ANOVA and, depending on the analysis, phase (number of 5-CSRTT training phases), region (DST and VST), or day (number of days in cocaine SA) together with housing condition was used as within-subject factor and line as between-subject factor. Acquisition of cocaine SA and the percentage of animals reaching the 60 cocaine infusions were assessed using the Kaplan–Meier survival analysis (O’quigley 1994). Upon confirmation of significant main effects, differences among individual means were further analyzed using the Student–Newman–Keuls’ test. Relationships between behavioral and SPECT parameters were examined using Pearson’s correlation coefficient. Data were analyzed using GraphPad Prism 9.0 (GraphPad Software, San Diego, CA). One rat in the RHA EE group died after the catheter implantation and was excluded from the analysis.

## Results

### Novelty-induced place preference (NIPP)

**Fig. 1:**
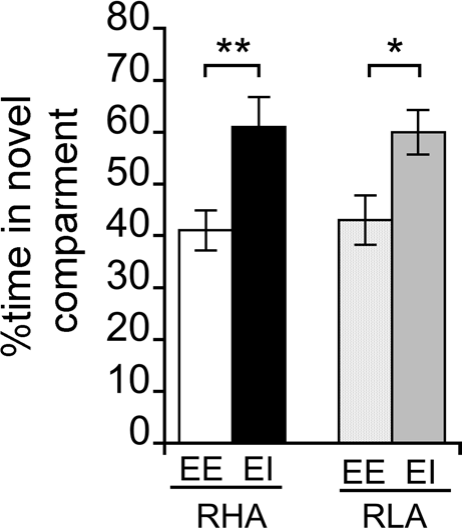
Percentage of total time spent in the novel compartment of the NIPP in RHA and RLA rats raised in EE or EI conditions. Data are shown as mean ± SEM. *p < 0.05 and **p < 0.01 significantly different from the respective EI group using a two-way ANOVA

A two-way ANOVA revealed a main effect of housing condition (*F*_1,51_ = 15.57, *p* < 0.001) but no main effect of line (*F*_1,51_ = 0.01, *p* > 0.05) and no line × housing condition interaction (*F*_1,51_ = 0.13, *p* > 0.05) on the time spent in the novel compartment. Irrespective of the line, EI rats spent more time in the novel compartment compared with EE rats (RHA EE vs. EI: *p* < 0.01; RLA EE vs. EI: *p* < 0.05). Noticeably, EE rats failed to show novelty place preference but a tendency to aversion, as indicated by a time spent in the novel compartment of circa 40% (Fig. 1).

### Five-choice serial reaction time task (5-CSRTT)

**Fig. 2:**
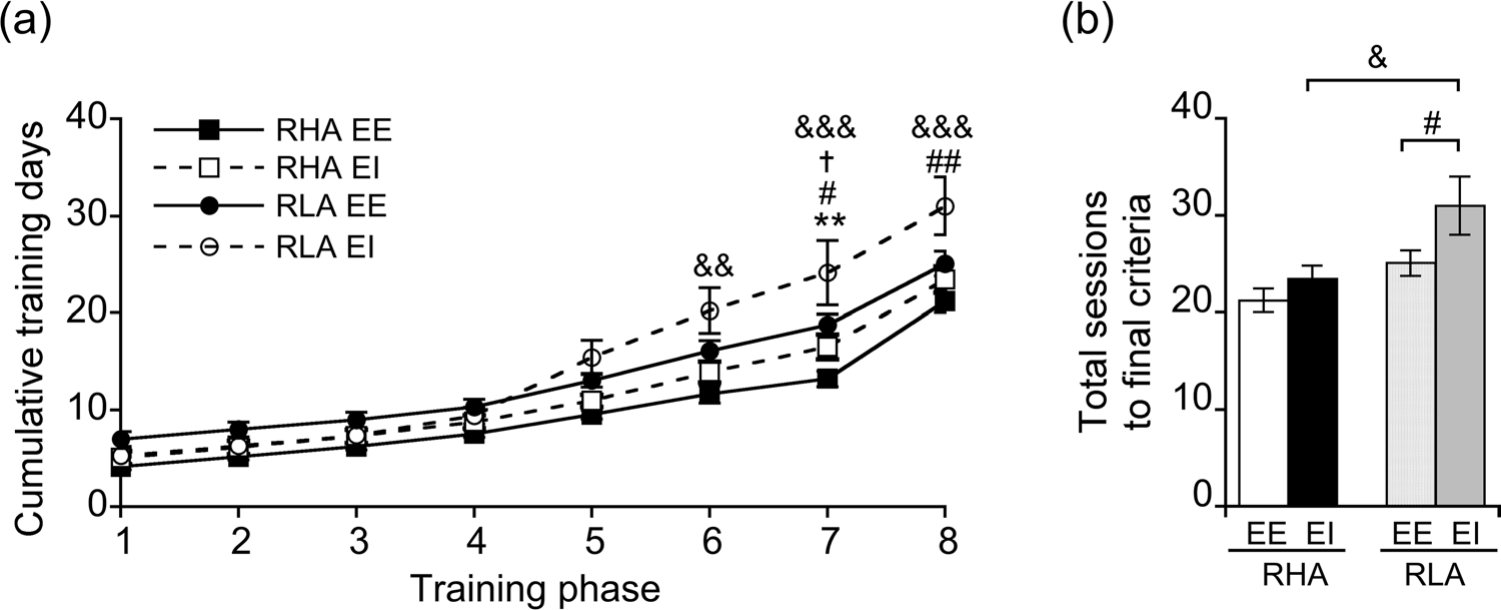
Acquisition of the 5-CSRTT in RHA and RLA rats raised in EE and EI conditions. **a** Mean cumulative number of sessions required to reach criterion to the next phase. **b** Total number of training days to reach the required learning criteria. Data are expressed as mean ± SEM. **p < 0.01 compared with the RHA EI group, #*p* < .05 and ##*p* < 0.01 compared with the RLA EI group, †*p* < 0.05 compared with the RLA EE group, &*p* < 0.05, &&*p* < 0.01, and &&&*p* < 0.001 compared with the RLA EI group using **a** mixed factorial ANOVA and **b** two-way ANOVA

For the 5-CSRTT acquisition, a mixed factorial ANOVA found a main effect of line (*F*_1,51_ = 15.76, *p* < 0.001), phase (*F*_7,357_ = 207.78, *p* < 0.001), line × phase (*F*_7,357_ = 5.35, *p* < 0.001), and housing condition × phase (*F*_7,357_ = 4.69, *p* < 0.001) interactions. Between-line comparisons showed that, regardless of the housing condition, RHAs acquired the 5-CSRTT faster when compared to RLAs (Fig. 2a). Moreover, post hoc analyses revealed that, while 5-CSRTT acquisition was similar in RHAs raised in EE and EI, RLAs raised in EE acquired the 5-CSRTT faster than RLA EI rats (Fig. 2a). When summing up the total number of training days needed for each group before impulsivity testing, a two-way ANOVA revealed a main effect of line (*F*_1,51_ = 9.11, *p* < 0.01) and housing condition (*F*_1,51_ = 4.62, *p* < 0.05) but no line × housing condition interaction (*F*_1,51_ = 0.97, *p* > 0.05). Between-line comparisons showed that no difference was found between RHA and RLA EE rats (*p* > 0.05), while RHA EI rats required less training days before testing as compared to RLA EI rats (*p* < 0.05; Fig. 2b). Moreover, post hoc contrasts revealed that, while no difference was found in RHAs (*p* > 0.05), RLA EE rats needed less training days to learn the task than their EI counterparts (*p* < 0.05; Fig. 2b).

**Fig. 3:**
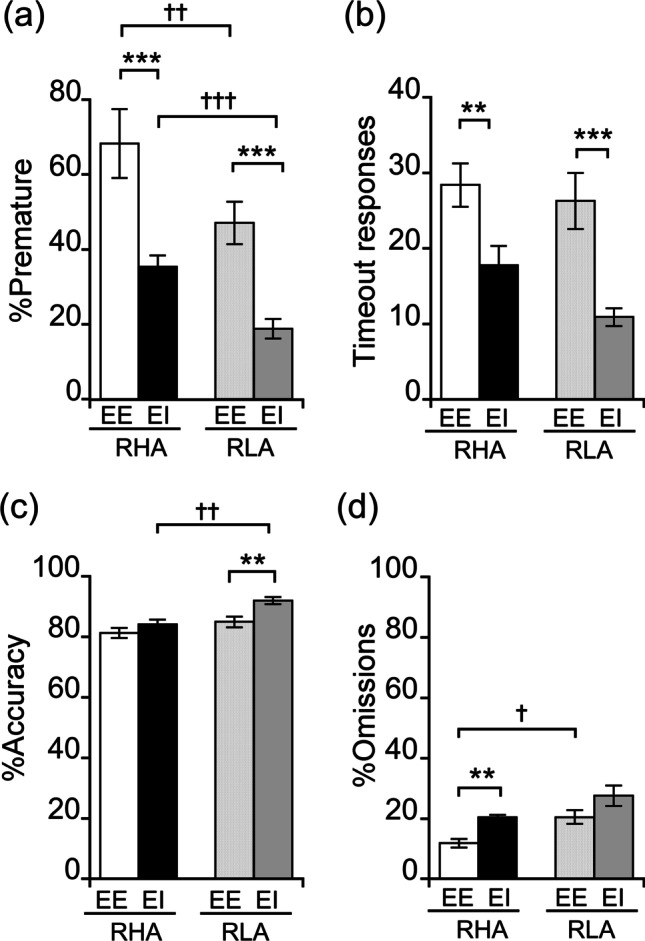
Performance of RHA and RLA rats raised in EE and EI conditions on 5-CSRTT performance. **a** Percentage of premature responses. **b** Number of timeout responses. **c** Percentage of accuracy. **d** Percentage of omissions. Data are expressed as mean ± SEM. Significantly different from EI rats at **p < .01 and ***p < .001 and significantly different from RLA rats at †*p* < .05, ††*p* < .01, and †††*p* < .001 using two-way ANOVA

When tested for premature responding in the 5-CSRTT, a two-way ANOVA revealed a main effect of line (*F*_1,51_ = 26.82, *p* < 0.001) and housing condition (*F*_1,51_ = 53.64, *p* < 0.001) but no line × housing condition interaction (*F*_1,51_ = 0.96, *p* > 0.05). Between-line comparisons showed that, irrespective of the housing condition, RHAs displayed higher percentage of premature responses than RLAs (RHA EE vs. RLA EE: *p* < 0.001; RHA EI vs. RLA EI: *p* < 0.01; Fig. 3a and Fig. S1). Post hoc contrasts also revealed that RHA and RLA EE rats displayed higher levels of premature responding than their EI counterparts (*p* < 0.001; Fig. 3a and Fig. S1). Additionally, and consistent with previous evidence in Wistar rats raised in EE vs. EI (van der Veen et al. 2015), both RHA and RLA EE rats made a higher number of TO responses as compared to their EI counterparts (Fig. 3b; two-way ANOVA; housing condition: *F*_1,50_ = 22.46, *p* < 0.001; line: *F*_1,50_ = 2.71, *p* > 0.05; line × housing condition: *F*_1,50_ = 0.76, *p* > 0.05).

Analyses of the percentage of accuracy (Fig. 3c) showed a main effect of line (*F*_1,51_ = 14.39, *p* < 0.001) and housing condition (*F*_1,51_ = 10.36, *p* < 0.01) but no line × housing condition interaction (*F*_1,51_ = 1.78, *p* > 0.05). Between-line analysis showed no between-line difference in the EE condition (*p* > 0.05), whereas, in the EI condition, RHA rats displayed lower accuracy than RLAs (*p* < 0.001; Fig. 3c). Moreover, post hoc contrasts revealed that, while no difference was found between EE and EI in RHA rats (*p* > 0.05), RLA EE rats displayed lower accuracy than their EI counterparts (*p* < 0.001; Fig. 3c). A main effect of line (*F*_1,47_ = 12.60, *p* < 0.001) and housing condition (*F*_1,47_ = 12.36, *p* < 0.001) but no line × housing condition interaction (*F*_1,47_ = 0.12, *p* > 0.05) was found in omissions. Between-line analyses showed that in the EE condition, RHA rats exhibited less percentage of omissions than RLAs (*p* < 0.05), whereas no such difference was detected in the EI condition (*p* > 0.05; Fig. 3d). Moreover, post hoc contrasts also revealed that RHA EE displayed lower percentage of omissions as compared to their EI counterparts (*p* < 0.01), whereas no difference was found in RLAs (*p* > 0.05; Fig. 3d).

### *SPECT imaging and the radioligand [*^*123*^* I]IBZM*

**Fig. 4:**
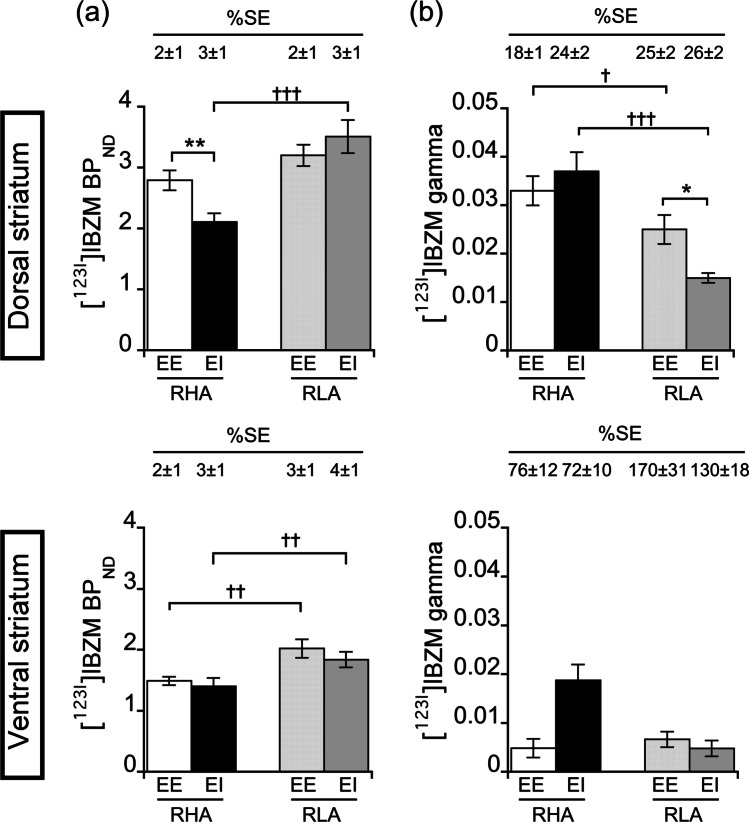
Mean **a** [^123^I]IBZM BP_ND_, an index of D_2/3_R availability, in the dorsal (top) and ventral (bottom) striatum and **b** [^123^I]IBZM gamma values, an index of AMPH-induced DA release, in the dorsal (top) and ventral (bottom) striatum in RHA and RLA rats raised in EE or EI conditions. Data are shown as mean ± SEM. *p < 0.05, **p < .01 and significantly different from the respective EI rats and †*p* < .0.05, ††*p* < 0.01, and †††*p* < .001 significantly different from the respective RLA rats using three-way ANOVA

A three-way ANOVA analysis of D_2/3_R availability in DST and VST, as indexed by [^123^I]IBZM BP_ND_, revealed a main effect of line (*F*_1,71_ = 34.42, *p* < 0.001), housing condition (*F*_1,71_ = 109.1, *p* < 0.001), and line × housing condition × region interaction (*F*_1,71_ = 5.09 *p* < 0.05). In DST, no between-line difference was found in EE rats (*p* > 0.05), whereas RHA EI rats displayed lower BP_ND_ in DST when compared with RLAs EI (*p* < 0.001; Fig. 4a). Moreover, post hoc contrasts also showed that, while RHA EE rats displayed higher BP_ND_ values when compared with RHA EI rats (*p* < 0.01), no difference was found between RLA EE and EI rats (*p* > 0.05; Fig. 4a). Regarding the VST, between-line comparisons revealed that, independently of the housing condition, RHAs displayed lower BP_ND_ values in VST than RLAs (*p* < 0.01; Fig. 4a). However, no difference in BP_ND_ was found in VST between EE and EI in either RHA or RLA rats (*p* > 0.05; Fig. 4a).

A representative time-activity curve showing the displacement of [^123^I]IBZM binding following AMPH in the DST of a RHA EE rat is shown in Fig. S2. A three-way ANOVA analysis of AMPH-induced DA release in DST and VST, as indexed by [^123^I]IBZM gamma, revealed a main effect of line (*F*_1,70_ = 30.59, *p* < 0.001), housing condition (*F*_1,70_ = 97,82, *p* < 0.001), line × housing condition (*F*_1,70_ = 4.58, *p* < 0.05), line × region (*F*_1,70_ = 13.78, *p* < 0.001), and housing condition × region (*F*_1,70_ = 6.31, *p* < 0.05) interactions. In DST, between-line comparisons indicated that, irrespective of the housing condition, RHAs showed higher gamma values, indicating a higher capacity to release DA in response to AMPH compared with RLA rats (RHA EE vs. RLA EE: *p* < 0.05; RHA EI vs. RLA EI: *p* < 0.01; Fig. 4b). Post hoc contrasts showed no difference in gamma between EE and EI in RHAs (*p* > 0.05), while RLA EE rats displayed higher gamma values compared with RLA EI rats (*p* < 0.05; Fig. 4b). As previously reported (Belles et al. 2020), [^123^I]IBZM gamma in DST was estimated with reasonably good precision, with %SE values lower than 30% (Fig. 4b). However, [^123^I]IBZM gamma in VST showed poor identifiability with %SE values exceeding 70% and 130% in RHA and RLA rats, respectively (Fig. 4b). [^123^I]IBZM gamma in VST was thus treated as unreliable and excluded from further analyses.

### Cocaine self-administration

**Fig. 5:**
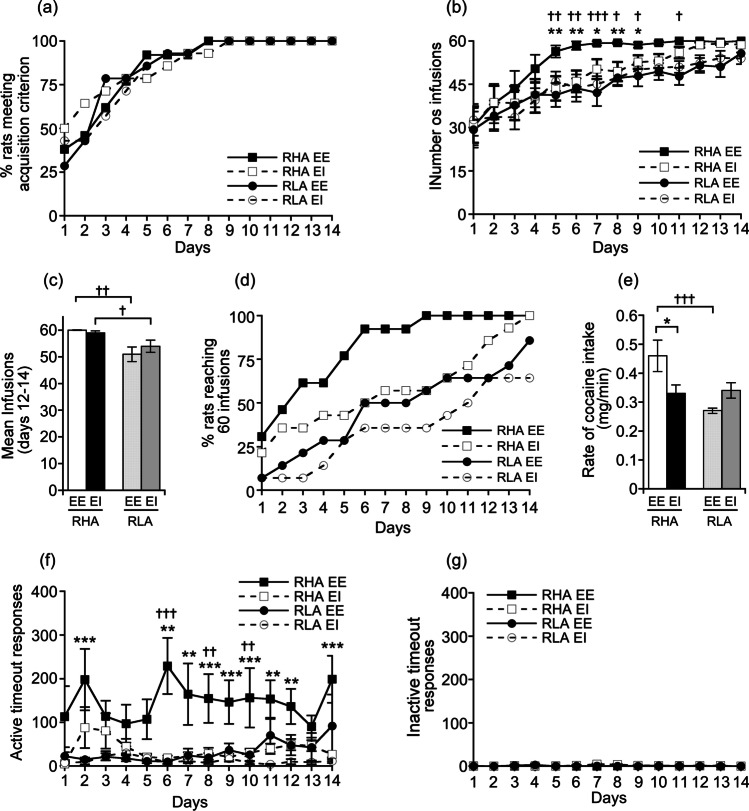
Acquisition and maintenance of cocaine SA in RHA and RLA rats raised in EE or EI conditions. **a** Cumulative percentage of rats that fulfilled cocaine SA acquisition criteria on each day of testing. **b** Number of cocaine SA infusions over 14 days of testing. **c** Mean number of cocaine infusions earned over the last 3 days of cocaine SA (sessions 12–14). **d** Cumulative percentage of rats that reached the maximum allowed of 60 infusions on each day of SA testing. **e** Mean rate of cocaine intake (in mg/min) over the 2 last days of testing (sessions 13–14). **f** Number of timeout responses on the active nose-poke hole per session over 14 days of testing. **g** Number of timeout responses on the inactive nose-poke hole per session over 14 days of testing. Data shown as mean ± SEM. *p < .05, **p < .01, and ***p < 0.001 significantly different from the respective EI rats and †*p* < .05, ††*p* < .01, and †††*p* < .001 significantly different from the respective RLA rats using **a** and **d** survival analysis; **b**, **f**, and **g** a mixed factorial ANOVA; and **c**, **e** two-way ANOVA

Irrespective of the housing condition, RHA and RLA rats acquired cocaine SA at a similar rate (*χ*^2^ = 0.02, df.1, *p* > 0.05; Fig. 5a), requiring a similar number of training sessions to meet the acquisition criteria (RHA EE: 3.00 ± 0.6 sessions; RHA EI: 2.86 ± 0.7 sessions; *F*_1,25_ = 0.02, *p* > 0.05; RLA EE: 3.00 ± 0.6 sessions; RLA EI: 3.14 ± 0.6 sessions; *F*_1,26_ = 0.03, *p* > 0.05). A mixed factorial ANOVA on the number of cocaine infusions over the 14 days of testing revealed a main effect of line (*F*_1,51_ = 7.98, *p* < 0.01) and day (*F*_13,663_ = 26.58, *p* < 0.001) but no main effect of housing condition (*F*_1,51_ = 0.93, *p* > 0.05), and no line × housing condition (*F*_1,51_ = 1.97, *p* > 0.05), line × day (*F*_13,663_ = 0.63, *p* > 0.05), housing condition × day (*F*_13,663_ = 0.73, *p* > 0.05), and line × housing condition × day (*F*_13,663_ = 0.78, *p* > 0.05) interactions. Post hoc analyses revealed that RHA EE rats displayed a higher number of cocaine infusions across sessions 5 to 9 and on session 11 as compared to RLA EE rats, while no between-line difference was found in the EI condition (Fig. 5b). Moreover, post hoc contrasts showed that RHA EE rats also exhibited a higher number of cocaine infusions across sessions 5 to 9 as compared to RHA EI rats, whereas no difference was found in RLAs (Fig. 5b). When looking at the mean number of cocaine infusions earned during the last 3 sessions of SA, a two-way ANOVA revealed a main effect of line (*F*_1,51_ = 14.70, *p* < 0.001) but no main effect of housing condition (*F*_1,51_ = 0.15, *p* > 0.05) and no line × housing condition interaction (*F*_1,51_ = 0.90, *p* > 0.05). Post hoc contrasts showed that, regardless the housing condition, RHAs self-administered more cocaine than RLAs during the last 3 days of testing (RHA EE vs. RLA EE: *p* < 0.01; RHA EE vs. RLA EE: *p* < 0.05; Fig. 5c). Analysis of the cumulative percentage of rats reaching the maximum of 60 infusions over the 14 days of testing showed that RHA EE also reached the 60-injection maximum faster than RLA EE (*χ*^2^ = 6.52, df.1, *p* < 0.01), while no difference was found between RHAs and RLAs in the EI groups (*χ*^2^ = 0.12, df.1, *p* > 0.05; Fig. 5d). Within-line comparisons indicated that RHA EE progressed to the 60 injections maximum faster than their EI counterparts (*χ*^2^ = 5.61, df.1, *p* < 0.01) while no difference between housing conditions was found in RLAs (*χ*^2^ = 0.32, df.1, *p* > 0.05; Fig. 5d). In keeping with these results, a two-way ANOVA of the total amount of cocaine consumed by the animals revealed a main effect of line (*F*_1,51_ = 8.03, *p* < 0.001) but no main effect of housing condition (*F*_1,51_ = 1.19, *p* > 0.05), and no line × housing condition interaction (*F*_1,51_ = 2.07, *p* > 0.05). Between-line comparisons showed that RHA EE rats consumed larger amount of cocaine than RLA EE rats (RHA EE: 302 ± 8 mg/kg vs. RLA EE: 249 ± 14 mg/kg; *p* < 0.01), whereas total cocaine intake in EI rats was similar (RHA EI: 271 ± 12 mg/kg vs. RLA EI: 254 ± 13 mg/kg; *p* > 0.05). In addition to progressing faster to the maximum of 60 infusions over the 14 days of testing, between-line comparisons revealed that RHA EE rats self-administered quicker the maximum allowed 60 cocaine infusions within 2-h SA sessions over the 2 last days of testing compared to RLA EE rats (two-way ANOVA; line: *F*_1,30_ = 3.75, *p* > 0.05; housing condition: *F*_1,30_ = 0.10, *p* > 0.05; line × condition interaction: *F*_1,30_ = 6.93, *p* < 0.01; RHA EE: 58 ± 6 min vs. RLA EE 90 ± 3 min; *p* < 0.01). In contrast, no between-line difference was found in the EI groups (RHA EI 78 ± 6 min vs. RLA EI 74 ± 5 min; *p* > 0.05). Moreover, post hoc contrasts also revealed that RHA EE were also quicker to self-administer the 60 infusions within sessions as compared to RHA EI rats (*p* < 0.05), whereas no difference was found in RLAs that reached the 60-infusion maximum (*p* > 0.05). In line with these findings, RHA EE rats exhibited a faster rate (in mg/kg) of cocaine intake over the 2 last days of testing as compared to RLA EE and RHA EI rats (two-way ANOVA; line: *F*_1,51_ = 15.47, *p* < 0.001; housing condition: *F*_1,51_ = 1.50, *p* > 0.05; line × housing condition: *F*_1,51_ = 8.03, *p* < 0.01; Fig. 5e).

RHA EE also displayed a significant higher number of active responses emitted during TO when compared to RLA EE, whereas no differences was detected between RHA and RLA EI rats (Fig. 5f; mixed factorial ANOVA; line: *F*_1,51_ = 28.73, *p* < 0.001; housing condition: *F*_1,51_ = 13.78, *p* < 0.001; day: *F*_13,534_ = 1.76, *p* < 0.05; line × housing condition: *F*_1,51_ = 4.75, *p* < 0.05; line × day: *F*_13,534_ = 1.85, *p* < 0.05; housing condition × day: *F*_13,534_ = 1.83, *p* < 0.05; line × housing condition × day: *F*_13,534_ = 0.99, *p* > 0.05; Fig. 5f). Moreover, post hoc analysis also revealed that RHA EE displayed higher number of active responses during TO as compared to RHA EI, whereas no difference was detected between RLA EE and EI rats (Fig. 5f). In the number of inactive responses during TO, a mixed factorial ANOVA showed a main effect of line (*F*_1,51_ = 4.21, *p* < 0.05) and a line × day interaction (*F*_13,661_ = 1.79, *p* < 0.05). However, post hoc contrasts did not reveal significant differences in the number of inactive responses during TO between lines (*p* > 0.05; Fig. 5g).

### *Relationships between [*^*123*^*I]IBZM binding measures and behavioral measures*

**Fig. 6:**
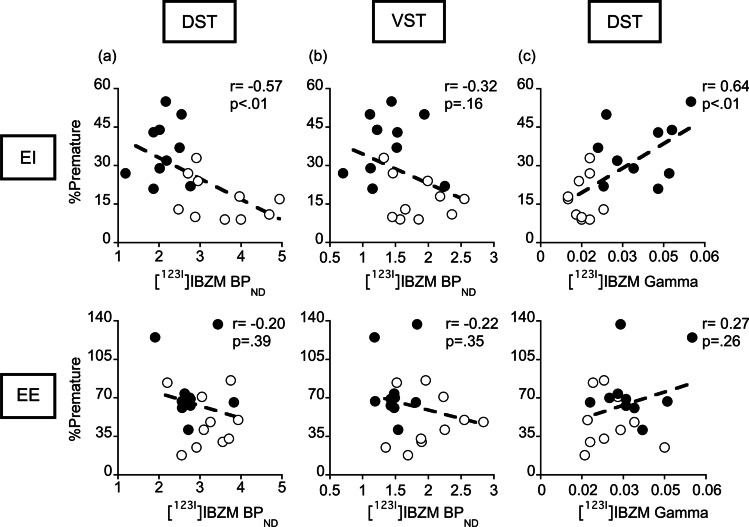
Relationships between premature responding and D_2/3_R-mediated indexes of DA signaling in EE and EI rats. RHA rats are depicted as closed symbols and RLA rats as open symbols. DST D_2/3_R availability (i.e., [^123^I]IBZM BP_ND_) was negatively correlated with impulsivity (**a**, top) in EI but not in EE housing conditions (**a**, bottom). No correlations were found between D_2/3_R availability in VST and impulsivity in EE and EI housing conditions (**b**). Additionally, AMPH-induced DA release (i.e., [^123^I]IBZM gamma) in DST was positively correlated with impulsivity (**c**, top) in EI but not in EE housing conditions (**c**, bottom). Correlations were tested using Pearson’s correlation coefficient

**Fig. 7:**
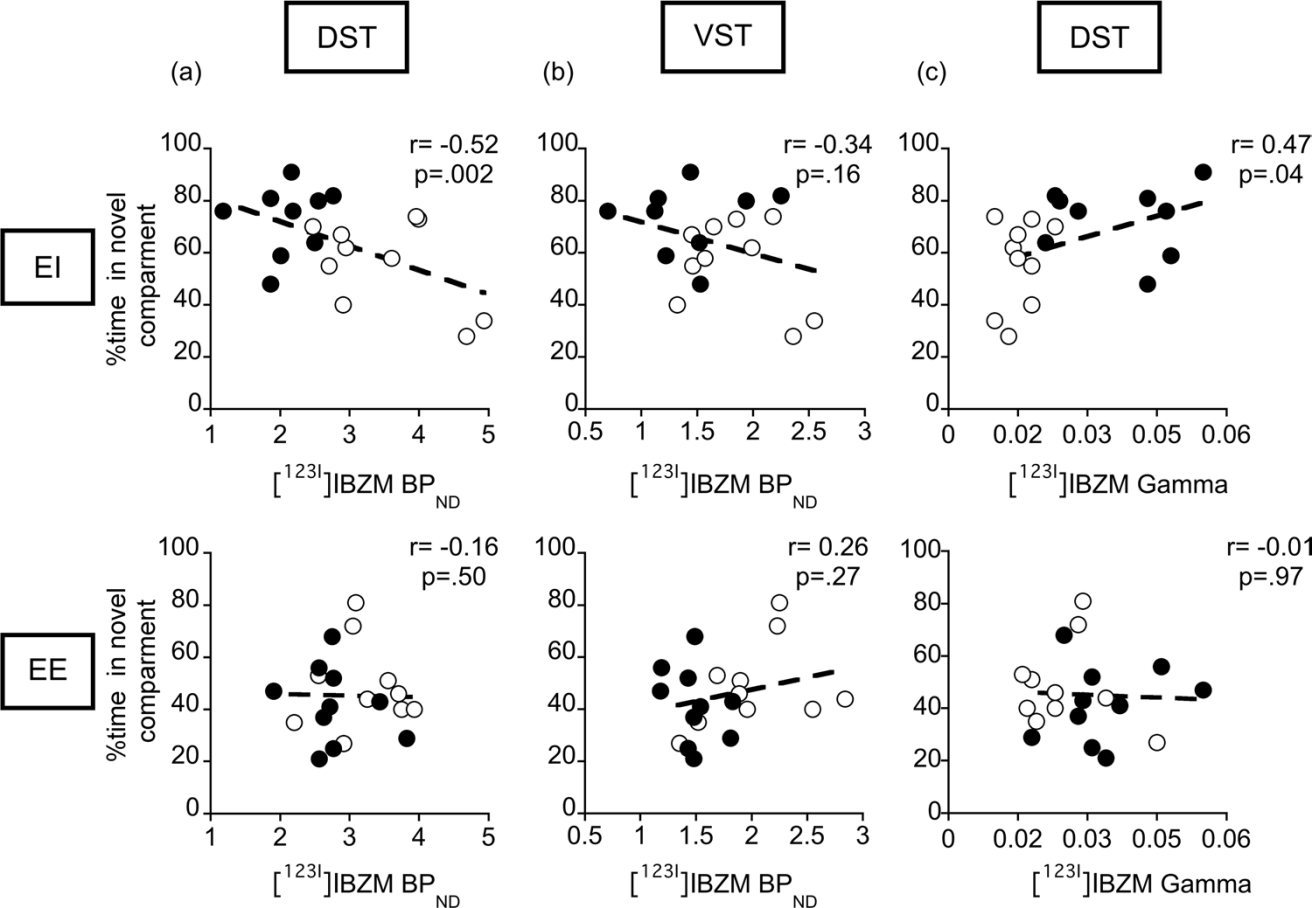
Relationships between novelty preference and D_2/3_R-mediated indexes of DA signaling in EE and EI rats. RHA rats are depicted as closed symbols and RLA rats as open symbols. DST D_2/3_R availability (i.e., [^123^I]IBZM BP_ND_) was negatively correlated with novelty preference (**a**, top) in EI but not in EE housing conditions (**a**, bottom). No correlations were found between D_2/3_R availability in VST and novelty preference in EE and EI housing conditions (**b**). Additionally, AMPH-induced DA release (i.e., [^123^I]IBZM gamma) in DST was positively correlated with novelty preference (**c**, top) in EI but not in EE housing conditions (**c**, bottom). Correlations were tested using Pearson’s correlation coefficient

In EI rats, premature responding was negatively correlated with D_2/3_R availability in the DST (i.e., BP_ND_; *r* =  − 0.57, *p* < 0.01, Fig. 6a), but positively correlated with AMPH-induced DA release (i.e., gamma; *r* = 0.64, *p* < 0.01; Fig. 6c), consistent with findings obtained in both lines reared in standard housing conditions (Belles et al. 2020). In contrast, no such correlations were found in EE rats (BP_ND_: *r* =  − 0.20, *p* = 0.39; Fig. 6a; gamma: r = 0.27, *p* = 0.26; Fig. 6c). Similarly, only in EI rats, novelty preference was negatively correlated with D_2/3_R availability in DST (*r* =  − 0.52, *p* = 0.002, Fig. 7a) and positively correlated with AMPH-induce DA release (*r* = 0.47, *p* = 0.04; Fig. 7c). Here again, no such correlations were found in EE rats (BP_ND_: *r* =  − 0.16, *p* = 0.50; Fig. 7a; gamma: *r* =  − 0.01, *p* = 0.97; Fig. 7c).

In EI rats, D_2/3_R availability in VST showed only weak correlations that failed to reach statistical significance with impulsivity (*r* =  − 0.32; *p* = 0.16; Fig. 6b) and novelty preference (*r* =  − 0.34; *p* = 0.16; Fig. 6b). No correlation was observed in EE rats (*r* =  − 0.22 and *p* = 0.35 for impulsivity; *r* =  − 0.26 and *p* = 0.27 for novelty preference; Fig. 7b).

As an exploratory analysis, with no correction for multiple comparisons, correlations between cocaine SA, behavioral, and SPECT variables were examined using Pearson’s correlation coefficient. No correlation was found in either EE or EI rearing conditions (Table S2).

## Discussion

This is the first study to concurrently measure the effects of early rearing environment on in vivo striatal D_2/3_R availability and psychostimulant-evoked DA release in relation to behavioral traits thought to predispose to drug abuse. When compared with EI, EE was associated with higher levels of impulsivity but a lack of novelty preference. While early rearing conditions had similar effects on both behavioral traits in RHA and RLA rats, EE and EI had differential effects on indices of DA function and predisposition to drug use in the two lines. When compared to EI, EE was related to higher D_2/3_R densities in the DST, but not VST, of addiction-prone RHAs only, and to higher striatal AMPH-induced DA release in RLAs only. Our results demonstrated that both impulsivity and novelty preferences were negatively correlated to DST D_2/3_R availabilities and positively correlated with AMPH-induced DA release in EI but not in EE, suggesting non-monotonic, environment-dependent, relationships between these addiction-related behavioral traits and indices of DA function that could offer a possible explanation for previous discrepant findings in the field. Moreover, the finding that RHA EE showed a faster rate of cocaine SA and elevated cocaine intake compared to their EI counterparts contrasts with the view that positive early-life experiences decrease the risk of drug abuse later in life (Puhl et al. 2012). Thus, exposure to EE is associated with high levels of impulsivity and may increase the reinforcing effects of psychostimulants in predisposed individuals (i.e., RHA rats). These findings indicate a potential role of epigenetic, environmental-dependent mechanisms as mediators of drug-induced brain neuroadaptations that underlie the drug addiction process.

Irrespective of the line, EE rats were more impulsive than EI rats, confirming previous results obtained in other rat strains (Dalley et al. 2002; Kirkpatrick et al. 2013; Zeeb et al. 2013). Independently of the line, and in contrast to EI rats, EE rats also showed a tendency toward novelty aversion, a result that corroborates recent findings in Sprague–Dawley (Vazquez-Sanroman et al. 2021). The reason for the lack of novelty preference in EE rats is unclear, but might be related to either the presence of novel objects in their home cage (Tanas et al. 2015; Vazquez-Sanroman et al. 2021) or to a faster habituation to novelty in EE animals (Modlinska et al. 2019; Schrijver et al. 2002; Zimmermann et al. 2001). Interestingly, the loss of novelty preference in EE rats is in line with previous research showing that EE dramatically blunted responding and incentive motivation for visual novelty (Cain et al. 2006).

While the impact of EE vs. EI on impulsivity and novelty preference was similar in RHA and RLA rats, EE had differential effects relative to EI on indices of DA signaling in DST in the two lines. In RHA rats, which exhibited low levels of DST D_2/3_R and a high magnitude of AMPH-induced striatal DA release in EI conditions, exposure to EE enhanced D_2/3_R availability but had no effect on evoked DA release. The opposite effects were observed in RLAs. Indeed, in RLA rats, which display high levels of DST D_2/3_R and low levels of AMPH-induced DA release in EI conditions, exposure to EE increased AMPH-induced DA release but had no effect on D_2/3_R. These results suggest that contrary to what was observed on behaviors, the impact of enriched rearing on DA functioning in DST is line-specific, environmental-, and baseline-dependent. Noticeably, [^123^I]IBZM BP_ND_ values used as in vivo indexes of D_2/3_R availabilities can be influenced by competition with basal levels of endogenous DA (Laruelle et al. 1995). The higher levels of [^123^I]IBZM BP_ND_ measured in RHA EE rats compared with their EI counterparts could thus represent either an increased D_2/3_R availability, a decreased in basal DA levels, or a combination of both. Previous studies on the effects of EE on endogenous DA levels have yielded inconsistent results. Some studies reported enhanced striatal DA concentration in EE vs. EI rats (Segovia et al. 2010), whereas others showed no change (Solinas et al. 2009; Zhu et al. 2005) or decreased (Bowling et al. 1993) basal levels of striatal DA in EE in relation to EI and standard housed animals. Therefore, we cannot exclude the possibility that the higher levels of D_2/3_R availability found in RHA EE vs. EI rats may have occurred as a result of a decreased basal concentration of endogenous DA. However, the finding of a differential effect of EE vs. EI on D_2/3_R in the DST of RHA and RLA rats is consistent with earlier work showing a line-dependent effect of EE on striatal D_2_R gene expression (Ravenelle et al. 2013). The failure to detect such a same effect in VST is unclear but may be related, at least in part, to a floor effect, given the lower levels of D_2/3_R, and therefore BP_ND_, measured in VST compared to DST.

Importantly, in previous studies, D_2/3_R availability and evoked DA release in the striatum were found to predict both impulsivity and novelty preference (Belles et al. 2020; Dalley et al. 2007). Noticeably, these previous findings were obtained from animals housed in standard conditions commonly found in laboratory animal housing facilities, where animals were housed in pairs in cages that consist of a single, small, open space with no environmental complexity, a relatively barren environment limited in physical and social stimulation Here, we showed that the relationships of reduced D_2/3_R availability and increased capacity to release DA in DST to impulsivity and novelty preference reported in standard conditions (Belles et al. 2020) can also be evidenced in impoverished but not in enriched housing conditions. These results thus suggest that the relationships between impulsivity, novelty preference, and indices of DA functioning are non-monotonic and that these behavioral traits likely also depend upon non-dopaminergic substrates. For instance, beyond DA, other neurotransmitters such as serotonin (5-HT) have also been linked to impulsivity (review in Dalley and Roiser 2012), with data indicating an opposing influence of different 5-HT receptor subtypes on this behavior (Fletcher et al. 2007; Robinson et al. 2008; Winstanley et al. 2004). On the other hand, early rearing conditions can also affect the concentration of 5-HT (Brenes et al. 2008; Kirkpatrick et al. 2014) and produce significant effects on the forebrain densities of different 5-HT receptor subtypes (Preece et al. 2004; Schiller et al. 2006). Besides changes in DA function, it is thus possible that early environment-induced alterations in 5-HT neurotransmission may also contribute to the effects of EE and EI on impulsivity and novelty preference.

Previous studies have reported conflicting results regarding the effect of environment on the propensity to acquire psychostimulant SA, showing either a decrease (Alvers et al. 2012; Gipson et al. 2011) or no effect (Hofford et al. 2014) of EE when compared to EI. Here we showed that, independent of the early environmental rearing conditions, RHAs and RLAs acquired cocaine SA at a similar rate, extending previous studies showing that both lines also showed a similar pattern of cocaine SA acquisition when reared in standard conditions (Dimiziani et al. 2019; Fattore et al. 2009). Similarly, irrespective of the line, we failed to reveal differences in the maintenance of cocaine SA between EE and EI rats, contrasting with previous studies reporting EE as a protective factor for drug maintenance (Bardo et al. 2001; Green et al. 2002). Nevertheless, in these studies, the impact of EE to decrease the acquisition and maintenance of drug-taking was dose-dependent, and at a cocaine dose similar to our (i.e., 0.4 mg/kg/inf), no environment-related difference in the number of infusions earned was found. Importantly, for ethical reasons, rats in our study were limited to 60 cocaine infusions/session. It is thus possible that with no such limitation, we could have been able to detect differences in the maintenance of cocaine SA between EE and EI animals. Yet, it has been shown that the propensity to acquire drug SA or to self-administer high levels of drugs such as alcohol (Giuliano et al. 2019, 2018) or cocaine (Deroche-Gamonet et al. 2004; Ducret et al. 2016) was not associated with the vulnerability to develop a compulsive pattern of drug-seeking/taking, which is a core feature of addiction. In such studies, drug addiction is operationalized by the (1) inability to refrain from drug-seeking when signaled as unavailable, (2) high breaking points during progressive ratio schedules of reinforcement, and (3) persistence of drug SA despite concurrent punishment with electric shock (Belin and Deroche-Gamonet 2012; Belin et al. 2008). Even though the housing environment did not affect SA acquisition and maintenance, “addiction-prone” RHA rats showed a remarkably higher rate of active responding during TO, increased their rate of cocaine intake faster over sessions, and reached the maximum allowed of 60 cocaine infusions within sessions quicker when raised in EE than in EI conditions. In contrast, no such environmental-related difference was observed in the “addiction-resistant” RLA rats. These findings indicate that cocaine may have greater reinforcing properties in EE relative to EI condition in the “addiction-prone” RHA but not in the “addiction-resistant” RLA rat line. If this is the case, such a line-specific effect suggests that neither early rearing conditions nor phenotype alone influences cocaine intake later in life but that both are risk factors that interact such that early environmental factors affect drug-taking behaviors in drug-vulnerable individuals. However, it is not clear from our data whether it is enriched rearing that increased cocaine-taking in RHAs or whether it is isolation rearing that decreased it. Previous data from our laboratory showed that, when raised in standard housing conditions, RHA rats display greater cocaine intake and a higher rate of active nose-poke responding relative to RLA rats (Dimiziani et al. 2019), comparable to the findings here of higher active nose-poke responding during TO in RHA EE relative to RHA EI, and to RLA EE and RLA EI. Taken together, this suggests that it is EI that may reduce the reinforcing effects of cocaine in RHA rats relative to the EE condition, lessening the between-line differences previously observed in standard housing conditions. A reduced cocaine reinforcing effect in RHA EI rats is consistent with previous evidence showing that EI rats are less sensitive to the reinforcing and motivational effects of cocaine than EE rats as indicated by lower breakpoints in a progressive ratio schedule of reinforcement in EI rats (Smith et al. 2009). Consistent with this hypothesis, when compared to enriched rearing, isolation rearing has been shown to decrease the rewarding effects of cocaine, AMPH, and morphine in conditioned place preference procedures (Bardo et al. 1995; Bowling and Bardo 1994; Coudereau et al. 1997; Schenk et al. 1983). Moreover, although activation of the DA mesostriatal system plays a central role in cocaine reward (Ikegami and Duvauchelle 2004), RHA EI rats exhibited reduced motivated behaviors for cocaine but similar increases in striatal AMPH-induced DA release compared to RHA EE. Thus, consistent with previous observations in mice (Solinas et al. 2009), the effects of the environment on the rewarding effects of cocaine in RHAs appear to be independent of different DA release in the striatum. The present findings, while providing further evidence for similar acquisition and maintenance of drug SA between EE and EI rats at high doses (Alvers et al. 2012; Bardo et al. 2001; Gipson et al. 2011; Green et al. 2002), also suggest that EE increases cocaine-taking behaviors in vulnerable individuals.

One limitation of our study was the inability to obtain reliable estimates of [^123^I]IBZM gamma in VST using the LSSRM. As previously reported (Belles et al. 2020), the unreliability of [^123^I]IBZM gamma estimates in VST is unclear but may be related to increases in radiotracer delivery coincident to the AMPH activation in this region. In such conditions, the amplitude of DA release (i.e., gamma) as estimated by the LSSRM is underestimated and unreliable (Normandin et al. 2012). A second limitation is the use of EI as a comparison condition to EE. Social isolation and a poor rearing environment can have profound effects on brain and behavior and, in particular, increase anxiety- and depressive-like behaviors, enhance spontaneous locomotor activity, and deteriorate learning and memory (Burke et al. 2017; Karkhanis et al. 2014; Solinas et al. 2010). Therefore, our findings do not preclude the possibility that EI stress-induced effects could have altered the behavioral and neurochemical mechanisms here investigated and consequently limited the value of EI as a comparison condition for the effects of EE. A third limitation of the study is that early environmental factors could also influence other behavioral traits that characterize the Roman rat lines. RHA and RLA rats are known to differ, among others, in their emotional responses to stress, in anxiety, working and reference memory, and Pavlovian aversive conditioning (review in Giorgi et al. 2019). Our findings thus do not preclude the possibility that, besides affecting impulsivity and novelty preference, EE and EI may have also altered other behavioral traits in Roman rats and that those changes may have contributed to the observed results.

Altogether, the present findings provide additional evidence for the role of environmental factors on drug-taking behaviors. While we further demonstrate an impact of EE on impulsivity and novelty preference (Kirkpatrick et al. 2013; Vazquez-Sanroman et al. 2021), this study additionally showed that these behavioral traits were associated with D_2/3_R availability and evoked DA release in DST only in impoverished animals, paralleling similar findings in animals housed in standard conditions (Belles et al. 2020). This finding revealed that the effects of environmental factors on addiction-related traits are likely not fully mediated by DAergic mechanisms and that other neurotransmitters such as 5-HT or glutamate could be implicated (review in Holmes et al. 2005). Moreover, our data suggested that early rearing EI could decrease the reinforcing effects of psychostimulants, attenuating, rather than potentiating, the propensity to drug abuse in vulnerable individuals. The present study further substantiates the view that the propensity to drug intake results not only from genetic but also epigenetic mechanisms associated with environmental factors during early life that are crucial to determining susceptibility to drug abuse later in life.

## Supplementary Information

Below is the link to the electronic supplementary material.

Supplementary file1 (DOC 1360 KB)
