# Supplementary material for: Early environmental enrichment and impoverishment differentially affect addiction-related behavioral traits, cocaine-taking, and dopamine D2/3 receptor signaling in a rat model of vulnerability to drug abuse
Source: Psychopharmacology (Berl). 2021 Aug 31;238(12):3543–57. doi: 10.1007/s00213-021-05971-z (PMC8629910; doi:10.1007/s00213-021-05971-z)
Supplement: Supplementary file 1 — Supplementary file1 (DOC 1360 KB) [file 213_2021_5971_MOESM1_ESM.doc]

**Psychopharmacology**

**Supplementary Information for**

**Early environmental enrichment and impoverishment differentially affect addiction-related behavioral traits, cocaine-taking and dopamine D2/3 receptors signaling in a rat model of vulnerability to drug abuse**

Lidia Bellés^1,2^, Andrea Dimiziani^1^, François R. Herrmann^3^, Nathalie Ginovart^1,2^

*^1^Department of Psychiatry, Faculty of Medicine, University of Geneva, Switzerland*

*^2^Department of Basic Neurosciences, Faculty of Medicine, University of Geneva, Switzerland*

*^3^Division of Geriatrics, Department of Rehabilitation and Geriatrics, Geneva University Hospitals, Switzerland*

Correspondence**:** Nathalie Ginovart, Departments of Psychiatry & Basic Neurosciences, Faculty of Medicine, Room E07-2550A, University of Geneva, Rue Michel Servet 1, CH-1211 Geneva, Switzerland. Tel: +41 22 379 53 66; E-mail: nathalie.ginovart@unige.ch

**This file includes:**

**Supplementary methods**

**Figures S1 to S2**

**Table S1 to S2**

**Supplementary references**

**Supplementary methods**

**Novelty-Induced Place Preference (NIPP)**

The compartments had different colored walls (black and white stripes vs. blue dots on white background) and distinct floor textures (rough in the black and white stripes compartment and smooth in the blue dots one). Each compartment contained two sets of 16 infrared light beams in the x-y axis to measure horizontal activity. The apparatus was controlled by a PC computer using ActiMot software (TSE Systems GmbH, Bad Homburg, Germany). The apparatus was cleaned between subjects using a disinfectant solution (DES-UR, Sineclor Hygiène SA, Switzerland).

**Five-Choice Serial Reaction Time Task (5-CSRTT)**

Rats were trained and tested using eleven identical operant chambers. Each chamber was housed inside of a ventilated, noise-attenuating box (Med Associates Inc., St. Albans, VT). One side of the chamber comprised a curve wall with five equally spaced nose poke apertures each equipped with a stimulus light. The other side contained a house light and food receptacle with a light connected to an external pellet dispenser. Entries into all apertures including the food receptacle were monitored by photocell infrared beams. MED-PC software (Med Associates Inc., St. Albans, VT) controlled and recorded all the experimental events. Dustless precision pellets were used as rewards (45 mg, Noyes dustless pellets, TestDiet®, St. Louis, MO). Training was conducted 5 days a week. Each session, consisting of 100 trials or 40-min duration, began with the delivery of a reward pellet and the illumination of the house light and the food receptacle light. To initiate the trial, the rat was required to nose-poke into the food receptacle. After an inter-trial interval (ITI), a light stimulus located into one of the five nose-poke apertures was pseudorandomly illuminated. A nose poke into the illuminated aperture was recorded as correct response and rewarded with a pellet. A response into the wrong aperture (an incorrect response), a response during the ITI (a premature response) or no response within the limited hold (an omission) were punished with a 5 sec timeout (TO) period during which all lights were turned off and no food was delivered. At the beginning, the training was carried out with long stimulus duration (30 sec) and limited hold (30 sec) and short ITI (2 sec), which were gradually adjusted based on the rat performances over 8 training phases. The final 5-CSRTT training phase (i.e., phase 8) parameters consisted in 1.5-sec stimulus duration, 5-sec limited hold, and 7-sec ITI with a criterion performance of ≥ 50 correct trials, ≥80% accuracy and ≤30% omissions. As previously reported by us and others (Belles et al. 2020; Moreno et al. 2010), differences with the original task (Bari et al. 2008) stemmed from difficulties for RLA rats to meet the ≤20% of omission criteria when the task demand increases. Compared to RHAs, RLAs display higher omissions when tested for impulsivity (Fig. 3d). This difference is unlikely to result from differences in their ability to learn the task, as RLAs perform equally well during the first learning phases of the task, and even exhibit greater accuracy than RHAs (Fig. 3c). Differences in speed processing or locomotor function are also likely not involved as both lines displayed similar latencies to correct responses (Table S1). One possibility though is that the higher omission rate measured in RLAs vs. RHA may arise from reduced motivation in RLAs under conditions of high effort demand as indicated by their higher latencies to collect rewards (Table S1). Considering the implication of DA in effort and reward value (Pasquereau and Turner 2013), with lower striatal dopaminergic tone associated with less willingness to work for reward (Hosking et al. 2015; Salamone and Correa 2002; Salamone et al. 2007), the lower striatal presynaptic DA tone found in RLA compared to RHA rats, may contribute to RLAs to be more sensitive to increases in response costs and explain their choice to waive reward (i.e., omissions) when task demand becomes high. Finally, impulsivity was tested in a session of 100 trials or 60-min duration, with the ITI extended to 9 sec; the stimulus duration was 1.5 sec and the limited hold 5 sec.

**SPECT imaging and the radioligand [^123^ I]IBZM**

*Radiotracer preparation*

^123^I radioiodide was purchased from Heider AG (Schöftland, Switzerland). [^123^I]IBZM was prepared as previously described (Tsartsalis et al. 2017). Briefly, a mixture containing 5 μl of BZM precursor, 2 μl of glacial acetic acid, 1 μl of 30% H_2_O_2_ and 10 mCi of carrier-free ^123^I sodium iodide in 0.05 M NaOH was incubated for 15 min at 68°C. Radiotracer was isolated by a linear gradient HPLC run (from 5% acetonitrile to 95% acetonitrile, 10 mM H_3_PO_4_ in 10 min). [^123^I]IBZM was eluted with 0.5 ml of 95% ACN, 10 mM H_3_PO_4_ and concentrated using a rotary evaporator, and the final product was diluted in saline prior to animal administration.

*SPECT imaging system*

Rats were scanned using the ultra-high-resolution multipinhole SPECT scanner (U-SPECT II, MiLabs, Utrecht, Netherlands, see (Deleye et al. 2013; Goorden et al. 2013). Briefly, U-SPECT II is a stationary system with three detectors (9.5-mm thick crystal NaI(Tl)). A collimator with 75 pinholes (pinhole size 1.0 mm) with a resolution of <0.8 mm and sensitivity >700 cps/MBq was used for focused rat imaging. The animal bed was translated in three dimensions for whole-body acquisitions in the defined FOV.

*SPECT imaging*

SPECT scans were performed under 2.0% isoflurane anesthesia. Radiotracer injection was performed via a tail vein catheter (24G). Body temperature was monitored during the scans and maintained at 37 ± 1 °C by means of a thermostatically controlled heating blanket. SPECT data acquisition started at the time of a bolus injection of 1 mCi [^123^I]IBZM for a total duration of circa 134 min. SPECT image reconstruction was performed using a pixel ordered subsets expectation maximization (POSEM, 0.4-mm voxel size, 4 subsets, 6 interactions) algorithm using MiLabs image reconstruction software. Radioactive decay correction was performed.

*SPECT data analysis*

Reconstructed SPECT images were processed using PMOD software v3.8 (PMOD Technologies Ltd, Zurich, Switzerland) as previously described (Belles et al. 2020). Dynamic images were smoothed using a 3D Gaussian kernel with 0.6 mm full width. Summed SPECT images were generated over the first 70 min and were automatically coregistred to the MRI atlas of the rat brain (Schiffer et al. 2006) using a Gaussian smoothing of 0.8 mm. Then, manual microadjustments were performed on the coregistered SPECT images. Transformation matrices were then applied to the SPECT dynamic images, mapping all rats into the same reference space. Time-activity curves (TACs) from the dorsal striatum (DST), ventral striatum (VST) and cerebellum were extracted from dynamic images using a customized region of interest (ROI) template. ROIs consisted of fixed-size circles placed bilaterally over the DST and VST. A single elliptic ROI was placed over the cerebellum cortex. TACs were analyzed using the linearized simplified reference region model (LSSRM; Alpert et al. 2003; Christian et al. 2006). Briefly, the LSSRM considers temporal perturbations in the radioligand specific binding caused by drug-induced changes in extracellular levels of endogenous neurotransmitter during a single-scan session. The LSSRM assumes that the physiologic steady state is not maintained throughout the experiment but allows the dissociation rate of the radioligand, k_2a_, to change over time in response to local variation in neurotransmitter concentration (k_2a_ = k_2_/[1+BP_ND_]), where k_2_ is the tissue-to-plasma efflux constant in the tissue region. Changes in BP_ND_ in activation studies are usually assumed to reflect changes in the concentration of available receptor sites for radioligand binding, and a decrease in BP_ND_ is assumed to reflect increased neurotransmitter release. The temporal change of k_2a_ (via a change in BP_ND_) is obtained by a time-dependent parameter k_2a_+gamma·h(t), where gamma represents the amplitude of the ligand displacement and the function h(t) describes a rapid change following task onset and dissipation over time. The exponential decay function h(t)=exp[−τ(t−T)] accounts for temporal variation in the model parameters, where τ controls the rate at which activation effects die away and T indicates the task initiation time. An increased k_2a_ reflects a decreased BP_ND_ for [^123^I]IBZM, caused by an AMPH-induced increase in DA release, which results in a positive value of gamma. In this study, T was set at 72-min post-radiotracer injection, at the time of AMPH administration. τ was set to 0 min^-1^ as the decrease in BP_ND_ caused by amphetamine (AMPH) administration is long-lasting, at least 3 hours (Ginovart et al. 2006; Laruelle et al. 1997) and is unlikely to recover during the 60 min of post-AMPH SPECT acquisition. The cerebellum was used as the reference region, because negligible densities of D_2/3_ receptors are found in this region (Hall et al. 1988).

**Cocaine self-administration**

*Intravenous catheter surgery*

Rats were anesthetized with 2.0% isoflurane and were implanted with an indwelling catheter (22 ga) (Instech Laboratories, Plymouth Meeting, PA, USA) according to a procedure previously described (Dimiziani et al. 2019). Briefly, under sterile conditions, rats were injected with 10 mg/kg of amikacin and 0.03 mg/kg buprenorphine (s.c.) as preoperative care. A silastic catheter was inserted into a jugular vein and anchored to the vein with suture. The distal end of the catheter run subcutaneously and exited in the midscapular region where it was secured with sutures to a sterile vascular access button (VAB, Instech Laboratories) covered with a protective aluminum cap (Instech Laboratories) designed to permit group housing. Then, rats were sutured and treated with 2% fusic acid cream (Leo Pharma, Ballerup, Denmark). Following surgery, the catheter was flushed with 0.1 ml of sterile heparinized saline (30U.I./ml) containing 67 mg/ml cefazolin for five days to minimize post-surgical infection. Rats were treated with 0.02 mg/kg of buprenorphine (s.c.) twice a day for 3 days to minimize post-surgical infection.

*Self-administration apparatus*

All sessions were conducted using eleven identical operant chambers each housed inside of a ventilated, noise-attenuating box (Med Associates Inc., St. Albans, VT). Each chamber was equipped with a house light and two nose-poke holes positioned on the left and on the right side of a lateral wall, 2.5 cm from the floor, and were equipped with a stimulus light and a photocell infrared beam. Intravenous cocaine infusions were delivered by a pump mounted outside the noise-attenuating box, though a tube connected to the rat’s catheter. The infusion tube was suspended into the chamber using a swivel mechanism, allowing the rat to freely move during session. MED-PC software (Med Associates Inc., St. Albans, VT) controlled and recorded all the experimental events.

**Supplementary Figures**

**Fig. S1**

**
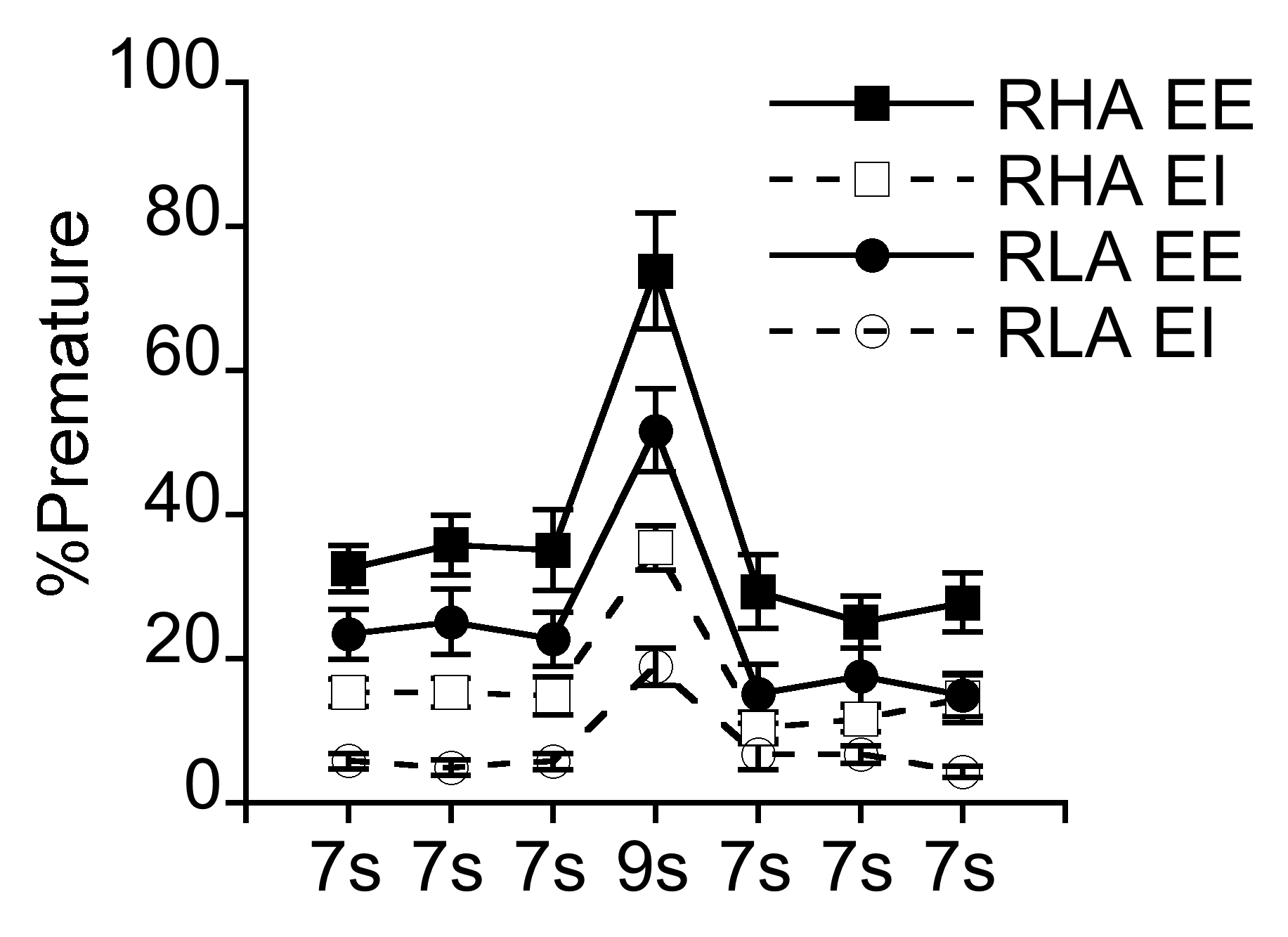
**

***Impulsivity on the 5-CSRTT. Shown are levels of impulsivity, expressed as a percentage of premature responses, during baseline sessions (ITI = 7 sec) and impulsivity test session (ITI = 9 sec) in RHA and RLA rats raised in EE or EI conditions.***

Irrespective of the housing condition, RHAs made more premature responses in both 7 and 9-sec ITI sessions. Furthermore, irrespective of the line, EE rats displayed more premature responses than EI rats during the 7 and 9-sec ITI sessions (housing condition: F_1,51_=49.80 *p*<.001; line: F_1,51_=97.56, *p*<.001; session: F_6,249_=37.03, *p*<.001; housing condition x line: F_1,51_= 3.28, *p*<.05; housing condition x session: F_6,249_=1.16 *p*>.05; line x session: F_6,249_=1.61 *p*>.05; housing condition x line x session: F_6,249_=1.26 *p*>.05). Data are expressed as mean±SEM using a mixed factorial ANOVA.

Fig. S2


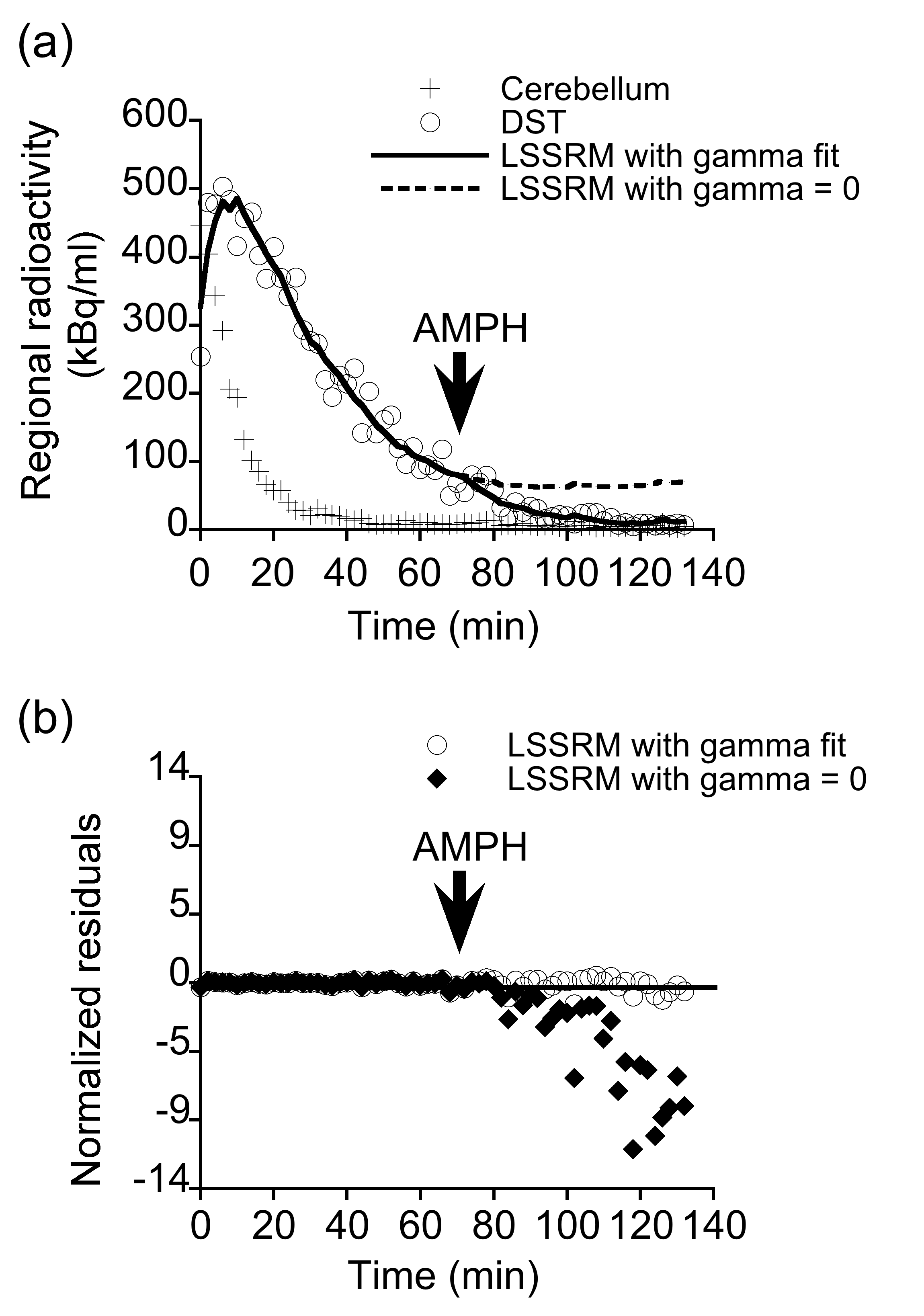


***A representative time-activity curve showing the displacement of [^123^I]IBZM binding following AMPH in the DST of a RHA EE rat.***

(a) Representative time-activity curve showing the displacement of [^123^I]IBZM binding following AMPH in the DST of a RHA EE rat. Time-activity data in cerebellum, used as reference region in the model, is represented by the plus symbol. The vertical arrow represents the time at which amphetamine (AMPH) was injected (i.e., 72 min post-radiotracer injection). The open black symbols represent the measured values in the DST, the solid line represents the fitted curve obtained according to the LSSRM with gamma fit, and the dashed line represents the fitted curve obtained according to LSSRM with gamma fixed to 0. (b) Normalize residuals (SPECT- model/SPECT) of the model fit with the gamma parameter (open symbols) and with gamma fixed to 0 (closed symbols) in the DST.

**Supplementary Tables**

**Table S1.** Behavioral performances RHA and RLA rats raised in EE or EI conditions on the 5-CSRTT.

| **Group** | **Accuracy (%)** | | | **Correct responses (%)** | | | **Omissions (%)** | | | **Premature responses (%)** | | | **Perseverative responses** | | | **Timeout responses** | | | **Latency to Correct Response (s)** | | | **Latency to Reward Collection (s)** | | |
| --- | --- | --- | --- | --- | --- | --- | --- | --- | --- | --- | --- | --- | --- | --- | --- | --- | --- | --- | --- | --- | --- | --- | --- | --- |
| **RHA EE** | 81.33 | ± | 0.02 | 69.11 | ± | 0.03 | 15.19 | ± | 0.04***^,††^ | 68.29 | ± | 9.19***^,†^ | 1.00 | ± | 0.38 | 28.42 | ± | 2.88** | 0.80 | ± | 0.03** | 1.38 | ± | 0.04^†††^ |
|  |  |  |  |  |  |  |  |  |  |  |  |  |  |  |  |  |  |  |  |  |  |  |  |  |
| **RHA EI** | 81.16 | ± | 0.02^†††^ | 66.57 | ± | 0.01 | 20.86 | ± | 0.01 | 35.36 | ± | 3.06^††^ | 1.64 | ± | 0.41 | 17.79 | ± | 2.55 | 0.91 | ± | 0.03 | 1.41 | ± | 0.04^†††^ |
|  |  |  |  |  |  |  |  |  |  |  |  |  |  |  |  |  |  |  |  |  |  |  |  |  |
| **RLA EE** | 85.72 | ± | 0.02*** | 64.52 | ± | 0.03 | 22.94 | ± | 0.03 | 47.11 | ± | 5.64*** | 1.71 | ± | 0.4 | 26.29 | ± | 3.74*** | 0.88 | ± | 0.03 | 1.89 | ± | 0.12 |
|  |  |  |  |  |  |  |  |  |  |  |  |  |  |  |  |  |  |  |  |  |  |  |  |  |
| **RLA EI** | 92.06 | ± | 0.01 | 64.43 | ± | 0.03 | 29.86 | ± | 0.04 | 18.86 | ± | 2.60 | 1.69 | ± | 0.35 | 10.86 | ± | 1.19 | 0.90 | ± | 0.05 | 1.87 | ± | 0.10 |

Data are mean ± SEM. Significantly different from EI rats at *p < 0.05, **p < 0.01 and ***p < 0.001 and significantly different from RLA rats at ^†^p < 0.05, ^††^p < 0.01 and ^†††^p < 0.001using a two-way ANOVA.

**Table S2.** Relationships between mean cocaine infusions and active timeout responses over the two last days of testing and indices of DA signaling, impulsivity, and novelty preference in EE and EI rats.

|  | | | **DST D_2/3_R availability** | **VST D_2/3_R availability** | **DST DA release** | **Impulsivity** | **Novelty preference** |
| --- | --- | --- | --- | --- | --- | --- | --- |
| **EE** | **Cocaine infusions** | Pearson's*r* | 0.197 | 0.058 | 0.303 | 0.257 | -0.039 |
|  |  | *P*-value | *0.405* | *0.807* | *0.194* | *0.274* | *0.871* |
|  | **Active TO responses** | Pearson's*r* | -0.284 | -0.430 | 0.356 | 0.388 | -0.242 |
|  |  | *P*-value | *0.225* | *0.058* | *0.124* | *0.091* | *0.304* |
| **EI** | **Cocaine infusions** | Pearson's*r* | 0.044 | 0.018 | 0.159 | 0.108 | 0.017 |
|  |  | *P*-value | *0.855* | *0.939* | *0.502* | *0.651* | *0.946* |
|  | **Active TO responses** | Pearson's*r* | 0.371 | -0.291 | 0.363 | 0.405 | 0.341 |
|  |  | *P*-value | *0.107* | *0.213* | *0.116* | *0.077* | *0.153* |

**Supplementary References**

Alpert NM, Badgaiyan RD, Livni E, Fischman AJ (2003) A novel method for noninvasive detection of neuromodulatory changes in specific neurotransmitter systems. Neuroimage 19: 1049-60.

Bari A, Dalley JW, Robbins TW (2008) The application of the 5-choice serial reaction time task for the assessment of visual attentional processes and impulse control in rats. Nat Protoc 3: 759-67.

Belles L, Dimiziani A, Tsartsalis S, Millet P, Herrmann FR, Ginovart N (2020) Dopamine D2/3 receptor availabilities and evoked dopamine release in striatum differentially predict impulsivity and novelty preference in Roman high- and low-avoidance rats. Int J Neuropsychopharmacol.

Christian BT, Lehrer DS, Shi B, Narayanan TK, Strohmeyer PS, Buchsbaum MS, Mantil JC (2006) Measuring dopamine neuromodulation in the thalamus: using [F-18]fallypride PET to study dopamine release during a spatial attention task. Neuroimage 31: 139-52.

Deleye S, Van Holen R, Verhaeghe J, Vandenberghe S, Stroobants S, Staelens S (2013) Performance evaluation of small-animal multipinhole muSPECT scanners for mouse imaging. Eur J Nucl Med Mol Imaging 40: 744-58.

Dimiziani A, Belles Ano L, Tsartsalis S, Millet P, Herrmann F, Ginovart N (2019) Differential involvement of D2 and D3 receptors during reinstatement of cocaine-seeking behavior in the Roman high- and low-avoidance rats. Behav Neurosci 133: 77-85.

Ginovart N, Galineau L, Willeit M, Mizrahi R, Bloomfield PM, Seeman P, Houle S, Kapur S, Wilson AA (2006) Binding characteristics and sensitivity to endogenous dopamine of [11C]-(+)-PHNO, a new agonist radiotracer for imaging the high-affinity state of D2 receptors in vivo using positron emission tomography. J Neurochem 97: 1089-103.

Goorden MC, van der Have F, Kreuger R, Ramakers RM, Vastenhouw B, Burbach JP, Booij J, Molthoff CF, Beekman FJ (2013) VECTor: a preclinical imaging system for simultaneous submillimeter SPECT and PET. J Nucl Med 54: 306-12.

Hall H, Farde L, Sedvall G (1988) Human dopamine receptor subtypes--in vitro binding analysis using 3H-SCH 23390 and 3H-raclopride. J Neural Transm 73: 7-21.

Hosking JG, Floresco SB, Winstanley CA (2015) Dopamine antagonism decreases willingness to expend physical, but not cognitive, effort: a comparison of two rodent cost/benefit decision-making tasks. Neuropsychopharmacology 40: 1005-15.

Laruelle M, Iyer RN, al-Tikriti MS, Zea-Ponce Y, Malison R, Zoghbi SS, Baldwin RM, Kung HF, Charney DS, Hoffer PB, Innis RB, Bradberry CW (1997) Microdialysis and SPECT measurements of amphetamine-induced dopamine release in nonhuman primates. Synapse 25: 1-14.

Moreno M, Cardona D, Gomez MJ, Sanchez-Santed F, Tobena A, Fernandez-Teruel A, Campa L, Sunol C, Escarabajal MD, Torres C, Flores P (2010) Impulsivity characterization in the Roman high- and low-avoidance rat strains: behavioral and neurochemical differences. Neuropsychopharmacology 35: 1198-208.

Pasquereau B, Turner RS (2013) Limited encoding of effort by dopamine neurons in a cost-benefit trade-off task. J Neurosci 33: 8288-300.

Salamone JD, Correa M (2002) Motivational views of reinforcement: implications for understanding the behavioral functions of nucleus accumbens dopamine. Behav Brain Res 137: 3-25.

Salamone JD, Correa M, Farrar A, Mingote SM (2007) Effort-related functions of nucleus accumbens dopamine and associated forebrain circuits. Psychopharmacology (Berl) 191: 461-82.

Schiffer WK, Mirrione MM, Biegon A, Alexoff DL, Patel V, Dewey SL (2006) Serial microPET measures of the metabolic reaction to a microdialysis probe implant. J Neurosci Methods 155: 272-84.

Tsartsalis S, Tournier BB, Aoun K, Habiby S, Pandolfo D, Dimiziani A, Ginovart N, Millet P (2017) A single-scan protocol for absolute D2/3 receptor quantification with [(123)I]IBZM SPECT. Neuroimage 147: 461-472.
